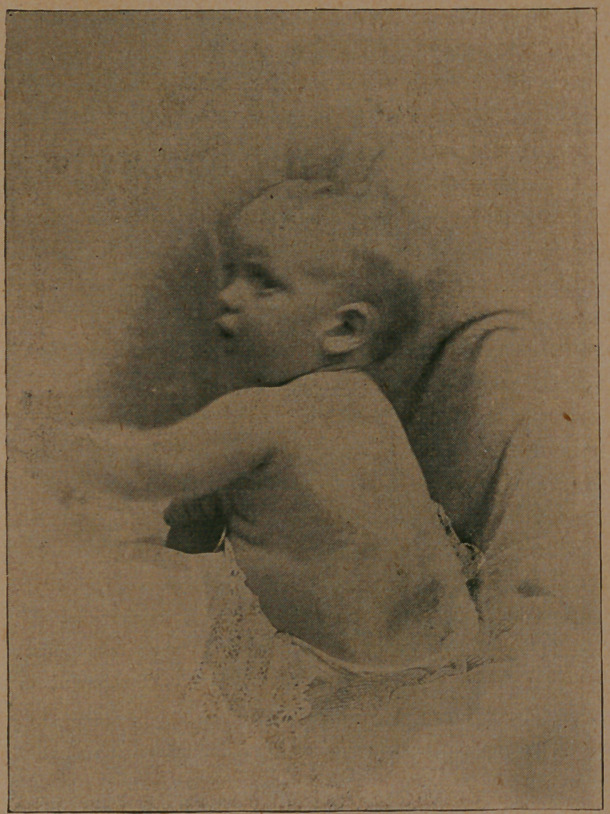# A Case of Spina-Bifida—Operation

**Published:** 1894-10

**Authors:** J. T. Feild

**Affiliations:** Fort Worth


For Texas Medical Journal.

### A CASE OF SPINA-BIFIDA—OPERATION.

BY J. T. FEILD, M. D., FORT WORTH.

[Read at Austin meeting Texas State Medical Association, April, 1894.]

THE USUAL course of these cases is towards death, and formerly they were but little interfered with. Now, however, with our improved methods of surgery, the outlook for these little patients is very much brighter.

Hoping to add something that may increase the interest in this class of patients, I make a brief report of the following case:

V. R., thirteen months old; affected with spina-bifida of lumbar region; tumor at birth size of hen's egg, gradually enlarging until it measured sixteen inches in circumference. The child also had talipes valgus. After a thorough understanding of the dangers attending the case, and my earnest recommendation to give the child the chance of an operation, the parents requested me to operate, which I did on the 21st of February, 1894, assisted by Drs. West, Duringer, Grammar and Burts, the latter giving the chloroform. I was very thorough in my antiseptics,, looking upon septic meningitis from a lack of thorough surgical cleanliness, as one of the greatest dangers; hence, I gave this my individual attention before, during and after the operation.

The operation was done by making two lateral flaps of the skin, which was very difficult, as it was so intimately blended with the dura, and very thin, being transparent. The dissection was continued until the opening in the vertebræ was reached. The child came near dying several times from too rough handling of the tumor. My attention would be called to it by Dr. Burts, and letting go of the tumor, the grave symptoms would disappear. The tumor was now punctured with a trocar and partially evacuated, elevating the hips and lowering the head, that there might not be too much cerebro-spinal fluid evacuated, thereby interfering with cerebral pressure. I incised the sac and emptied it of its contents. After convincing myself that there were no nerves in or spread out upon its walls, I sutured it as deeply in the spinal canal as possible, using the coblers' stitch; then just external to this row of sutures, transfixed with double ligatures, and, after securely tying, cut away the sac which was composed of the meninges. The approximate muscular and apon-neurotic tissues were drawn together and over the pedicle by a number of sutures. All the sutures used up to this time were catgut. The flaps made by the skin found discolored from a lack of circulation were removed. By silk worm sutures and firm pressure from each side I was enabled to close the wound, thus completing the operation. The wound was dressed in the usual antiseptic manner, being careful to seal hermetically the lower portion of the dressing by adhesive plaster to prevent contamination of the wound by the child's discharges.

First dressing removed on the eighth day; wound doing well; every indication of perfect union. The wound was dressed about every fourth day until the twenty-first day, when all dressing were left off, recovery being complete.

I submit herewith a photograph of the child before and after the operation. —*From Transactions T. S. M. A., 1894.*

## Figures and Tables

**Figure f1:**
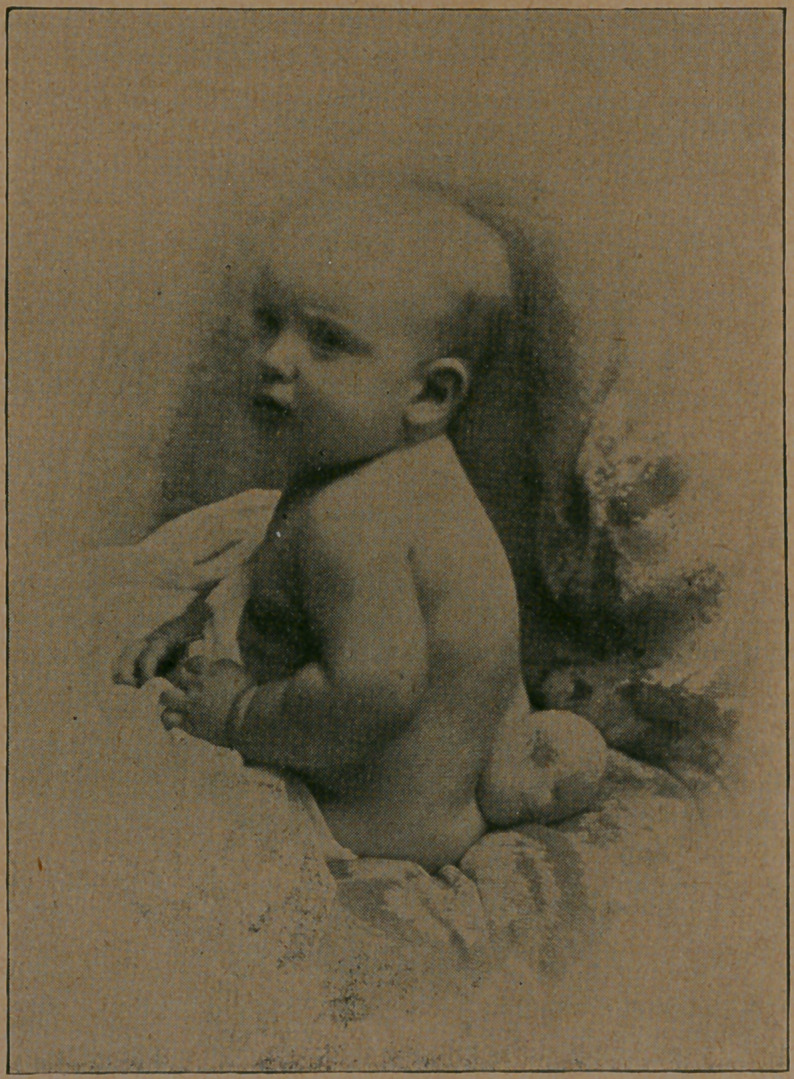


**Figure f2:**